# 'My dreams are shuttered down and it hurts lots’–a qualitative study of palliative care needs and their management by HIV outpatient services in Kenya and Uganda

**DOI:** 10.1186/1472-684X-12-35

**Published:** 2013-10-07

**Authors:** Lucy Selman, Victoria Simms, Suzanne Penfold, Richard A Powell, Faith Mwangi-Powell, Julia Downing, Nancy Gikaara, Grace Munene, Irene J Higginson, Richard Harding

**Affiliations:** 1 King’s College London, Department of Palliative Care, Policy and Rehabilitation, Cicely Saunders Institute, Bessemer Road, London SE5 9PJ, UK; 2 HealthCare Chaplaincy, 307 East 60th Street, New York, NY 10022, USA; 3 Formerly of the African Palliative Care Association, PO Box 72518, Kampala, Uganda; 4 International Palliative Care Initiative, Open Society Foundations, 400 West 59th Street, New York, NY 10019, USA; 5 Kenya Hospices and Palliative Care Association, P.O Box 20854, 00202, Nairobi, Kenya

**Keywords:** Kenya, Uganda, HIV, Distress, Experience, Pain, Drug availability, Qualitative research

## Abstract

**Background:**

Despite the huge burden of HIV in sub-Saharan Africa, there is little evidence of the multidimensional needs of patients with HIV infection to inform the person-centred care across physical, psychological, social and spiritual domains stipulated in policy guidance. We aimed to describe the problems experienced by people with HIV in Kenya and Uganda and the management of these problems by HIV outpatient services.

**Methods:**

Local researchers conducted in depth qualitative interviews with HIV patients, caregivers and service staff at 12 HIV outpatient facilities (6 in Kenya, 6 in Uganda). Interview data were analysed thematically.

**Results:**

189 people were interviewed (83 patients, 47 caregivers, 59 staff). The impact of pain and symptoms and their causes (HIV, comorbidities, treatment side-effects) were described. Staff reported that effective pain relief was not always available, particularly in Kenya. Psychosocial distress (isolation, loneliness, worry) was exacerbated by stigma and poverty, and detrimentally affected adherence. Illness led to despair and hopelessness. Provision of counselling was reported, but spiritual support appeared to be less common. Neither pain nor psychosocial problems were routinely reported to service staff. Collaboration with local hospices and income-generation activities for patients were highlighted as useful.

**Conclusions:**

The findings demonstrate the multiple and interrelated problems associated with living with HIV and how psychosocial and spiritual distress can contribute to 'total pain’ in this population. In line with the palliative care approach, HIV care requires holistic care and assessment that take into account psychological, socioeconomic and spiritual distress alongside improved access to pain-relieving drugs, including opioids.

## Background

In 2010, 22.9 million people in sub-Saharan Africa were living with HIV, 68% of the global disease burden [1]. In the same year, 1.2 million people died of AIDS and 1.9 million adults and children became infected with the illness [1,2].

HIV in Africa is associated with significant morbidity and poor quality of life [3-6]. High pain prevalence, caused by the underlying disease progression [7,8], comorbidities [9,10] and opportunistic infections [11], have been reported throughout the disease trajectory [11-13], irrespective of antiretroviral therapy (ART) receipt [7,14]. In Tanzania, a study of 731 patients attending HIV outpatient care with ART access found that 41.4% of patients were experiencing pain [15], and of 250 people in Rwanda living with HIV/AIDS, 43% required pain relief and symptom management [16].

Other physical and psychological symptoms are also highly prevalent. Peltzer and Phaswana-Mufaya [17] surveyed 607 people with HIV in South Africa and found a mean of 26.1 symptoms (SD 13.7), the most prevalent being headaches (79%), fever (69%), thirst (68%), fatigue (67%) and weakness (66%). Rates of psychological symptoms, such as fear/worry (59%), depression (55%) and anxiety (50%) were also high. Similarly, a survey of southern African HIV patients found prevalence rates of 45% for fear/worry, 40% for depression and 27% for anxiety (n = 743) [18]. Freeman et al. [19] found a point prevalence rate for mental disorder of 43.7% among 900 HIV-infected patients in South Africa.

HIV also presents a unique set of spiritual and existential challenges to patients as they confront aspects of living with a progressive, incurable disease that is highly stigmatized. In a study of 285 patients receiving palliative care in South Africa and Uganda (over 80% of whom had HIV infection), Selman et al. [20] found that 21-58% experienced spiritual distress. The symptom burden of HIV is compounded by poverty. In the survey by Peltzer and Phaswana-Mufaya, 47% of HIV patients reported sometimes and 12% reported often having insufficient food in the past 12 months, and this was associated with higher symptom frequency [17].

Owing to this growing body of evidence demonstrating the prevalence of multidimensional problems among HIV patients, international policy guidelines stipulate that a holistic, person-centred palliative care approach should be integral to HIV care throughout the disease trajectory [21]. However, quantitative studies are insufficient to throw light on the impact of HIV on patients’ everyday lives and the ability of HIV outpatient services to meet those needs. Achieving a true understanding of the illness experience requires qualitative research methods to explore patients’ experiences from an emic perspective [22], yet qualitative studies exploring the needs and experiences of HIV patients in the region are lacking [23,24]. In particular, the multidimensional burden associated with living with HIV and how HIV services respond to this burden has not been described. Evidence in this area is essential to model appropriate services that meet mandated guidelines for integrated HIV and palliative care.

This study aimed to describe the palliative care needs of HIV outpatients and the management of multidimensional problems by HIV outpatient services in Kenya and Uganda, to inform the provision of HIV care and support in sub-Saharan Africa.

## Methods

### Study design

Qualitative semi-structured interviews were conducted with HIV outpatients, informal caregivers and healthcare staff during the PEPFAR (President’s Emergency Plan for AIDS Relief) Care and Support public health evaluation (Phase 2) [25].

### Setting

The study was set in Kenya and Uganda. The Kenyan and Ugandan contexts represent the modern HIV epidemic, with moderate to high coverage of ART (61% in Kenya, 47% in Uganda [1]), stable prevalence [26] (6.3% in Kenya, 6.5% in Uganda [27]), and relatively good access to healthcare in urban areas [28].

### Sites and participants

Six facilities were selected in each country (see Table 1 for facility characteristics). These facilities were the largest of the 60 that were randomly selected from the c.1200 facilities receiving PEPFAR HIV Care and Support funding in Kenya and Uganda during Phase 2 of the PEPFAR evaluation [25]. Facility exclusion criteria were offering paediatric-only care and inaccessibility (e.g. insecure, no road access).

**Table 1:** Characteristics of facilities

**Facility**	**Type**	**Location**	**Patients in 2007**	**New patients in 2007**	**Access to strong opioids**	**Access to weak opioids**
A	HIV clinic of public hospital	Kenya, Rural	Unknown	246	No	No
B	HIV clinic of public hospital	Kenya, Urban	3031	377	No	No
C	HIV clinic of public hospital	Kenya, Urban	4334	463	No	No
D	NGO	Kenya, Urban	1126	796	No	Yes
E	HIV clinic of public hospital	Kenya, Urban	4963	547	No	No
F	HIV health centre	Kenya, Rural	5975	422	No	No
G	HIV clinic in mission hospital	Uganda, Rural	2075	79	By referral	Yes
H	HIV NGO	Uganda, Urban	4772	401	Yes	Yes
J	HIV NGO	Uganda, Urban	Missing	Missing	No	Yes
K	HIV clinic/research centre in public hospital	Uganda, Urban	9698	828	No	Yes
L	HIV clinic/research centre in public hospital	Uganda, Urban	7062	5774	Yes	Yes
M	HIV clinic in public hospital	Uganda, Urban	5602	683	Yes	Yes

In both countries, all facilities had full time doctors. Strong opioids were available at half of the Ugandan facilities but none of the Kenyan facilities. In Kenya, only two facilities (A, E) had any specialist spiritual care staff and only two (D, F) had any specialist psychological support staff. In Uganda, three facilities (G, K, M) had spiritual care staff and all facilities had specialist psychological support staff. For further details of the participating services, see Phase 2 reports [29,30].

We aimed to recruit seven patients, three caregivers and five staff at each site, providing an overall target of 84 patients, 36 caregivers and 60 staff members across both countries. Eligible participants for the patient interviews were adult patients (at least 18 years old) diagnosed with HIV infection who had been under the facility’s care for at least six weeks and were not involved in the Phase 1 cohort study also conducted during the PEPFAR study (not reported here) [25]. Patients were recruited purposively by gender, age and ART use to represent a range of experiences and perspectives. Patient participants were asked for consent to approach an identified adult informal caregiver (i.e. family member/friend who provided support). For staff recruitment, purposive sampling ensured a variety of designations with direct patient contact.

### Ethical approval

Ethical approval to undertake the study was obtained from the Ugandan National Council for Science and Technology, Kenyan Medical Research Institute and King’s College London Research Ethics Committee.

### Data collection

Interviews were conducted between February and September 2008. Interviews with patients and caregivers followed interview schedules covering history of accessing the facility, contact with service providers (including positive/negative aspects and drug access), principle problems/needs, and the nature/content of clinical encounters. The staff interview schedule covered role and experience, patients’ access to the facility, the nature/content of clinical encounters, referral, training, components of care, and facility strengths, weaknesses and challenges.

Interview schedules, information sheets and consent forms were translated from English into local languages (Kiswahili, Dholuo, Runyakitara and Luganda in Uganda; Kiswahili and Dholuo in Kenya) independently by two local researchers. Each version was back translated by a third researcher, with any discrepancies discussed by the research group to agree upon translation.

Interviews with staff members, patients and caregivers were conducted in private (usually in consulting rooms at the facility) and digitally recorded. All participants gave informed consent to participate following provision of an information sheet and consent form, which were read aloud to the interviewee for illiterate prospective participants.

Interview recordings were transcribed into the language in which they were conducted. Those transcripts not in English were translated independently into English by two translators, either study researchers or linguistics experts from a local academic institution. A team of three then reconciled the two independent translations, referring back to the recorded interview if necessary, and agreed a final version.

### Analysis

Anonymised patient, caregiver and staff transcripts were analysed concurrently using thematic content analysis [31,32] to enable multiple perspectives on each theme. The research team included the four interviewers (two in Uganda and two in Kenya), the two local principal investigators, who were experienced palliative care clinicians, and the three social scientist palliative care researchers at King’s College London. The team was divided into three sub-groups for the purposes of analysis. Each group of three members (in Kenya, Uganda and the UK) independently created a coding frame: in each group, every researcher coded eight randomly selected interviews (three with a patient, three with a staff member and two with a caregiver) and then met to agree on a coding frame by discussion, comparison and consensus. The three teams met to explore cross-cultural differences and similarities in coding and combine the strengths of each country-level frame to generate a unified frame. The final coding frame reflected local understandings and expertise while enabling standardised and comparable analysis that met the research aims. Each code was reviewed for internal consistency and given an agreed definition to ensure it was applied using a standard meaning by each researcher. The definitive coding frame was then applied to the entire dataset using NVivo v9 software, with a random sample of 12 transcripts independently checked by other team members to ensure the coding frame was applied consistently.

Participants’ age, gender, household location, family size, profession (for staff), relationship to patient (for caregivers) and whether they were receiving ART (for patients) were imported into NVivo, and sample characteristics described.

## Results

Eighty three patients, 47 caregivers and 59 staff were interviewed, giving a total of 189 participants (98 in Kenya; 91 in Uganda). Participant characteristics are presented in Table 2.

**Table 2:** Participant characteristics (N = 189)

**Patients (N = 83)**			
**Characteristic**	**Kenya (N = 42)**	**Uganda (N = 41)**	**Total (N = 83)**
Female N (%)	28 (67)	20 (49)	48 (58)
Mean age (range, median)	35 (20-56, 34)	37 (18-61, 37)	36 (18-61, 37)
Receiving ART N (%)	29 (71)	28 (68)	57 (69)
Mean household size (range)	4.7 (1-10)	6.7 (1-13)	5.7 (1-13)
Location			
Rural	23	7	30
Peri urban	9	18	27
Urban	9	16	25
**Care givers (N = 47)**			
**Characteristic**	**Kenya (N = 26)**	**Uganda (N = 21)**	**Total (N = 47)**
Female N (%)	14 (54)	14 (67)	28 (60)
Mean age (range, median)	40 (20-72, 37)	29 (18-52, 27)	35 (18-72, 32)
Mean household size (range)	5.7 (2-10)	6.5 (2-11)	6 (2-11)
Location			
Rural	14	8	22
Peri urban	7	7	14
Urban	5	6	11
Relationship to patient^a^			
Mother	4	2	6
Father	1	0	1
Sister	1	1	2
Brother	3	4	7
Daughter	2	3	5
Son	2	0	2
Wife	2	6	8
Husband	6	1	7
Aunt	3	1	4
Friend	1	3	4
Cousin	1	0	1
**Staff members (N = 59)**			
**Characteristic**	**Kenya (N = 30)**	**Uganda (N = 29)**	**Total (N = 59)**
Designation			
Nurses	5	6	11
Counselors	1	8	9
Medical officers	2	5	7
Clinical officers	4	2	6
Nurse counselors	2	3	5
Nutritionists	4	0	4
Doctors	2	2	4
Community nurses/workers	4	0	4
Social workers	1	1	2
Lab technologists	1	1	2
Nursing officers	1	1	2
Others	3	0	3
Mean time working at the facility (range, median)	5 years (2 months to 26 years, 2 years)	5 years (2 months to 24 years, 4 years)	5 years (2 months to 26 years, 3 years)

^a^ Sometimes these are categorical terms rather than blood relationship, for example 'father’ is also used to designate one’s father’s brother.

There were some differences between the participants in Kenya and Uganda. In Uganda, the sample of patients was 51% male (n = 21), median age 37, while in Kenya, 67% (n = 28) were female, median age 34. In Uganda, two thirds of carers (n = 14) were women, while in Kenya just over half of carers (n = 14) were women. Mean household sizes were larger in Uganda than Kenya. In both countries, the majority of patients were receiving ART (68%, n = 28 in Uganda, 71% (n = 29) in Kenya).

The staff interviewed represented many disciplines. In Uganda, staff consisted of seven counsellors, five clinical officers, five nurses, three nurse counsellors, two doctors, two medical officers and five other grades; in Kenya, six clinical officers, four nurses, four nutritionists, two nurse counsellors, two doctors, two community nurses and ten staff of other grades. The median time staff had worked at the facility was four years in Uganda (range two months to 24 years) and two years in Kenya (range two months to 26 years).

Patients’ multidimensional problems and facilities’ management of those problems emerged as central themes. Four subthemes emerged: pain and physical symptoms, psychosocial distress, spiritual distress, and the interconnected nature of patient problems. For the first three subthemes, descriptions of patient problems are followed by data regarding their management.

When quoting participants, unique identification codes are used as follows: P (patient), C (caregiver) or S (staff) followed by an identifying number and facility code (see Table 1).

1. Pain and physical symptoms

a. Description of pain and symptoms

Experiences of pain and other symptoms caused by HIV as well as by comorbidities were described by patients, regardless of ART status:

*'My chest is painful and uncomfortable; I am coughing and producing very black/dark saliva and I am wondering why… [And] I have painful joints and especially when I am sleeping… whenever I lie down it becomes difficult to rise up.’* P3 facility E, male, age 38, not on ART

Patients described symptoms associated with neuropathic pain, such as peripheral pain in the feet ('*[it] feel[s] like I have stayed in cold water for a long time.*’ P4 facility E, male, age 47, on ART).

The side effects of treatment were perceived to cause pain and other symptoms, although not for all patients:

*'When I started taking the drug [ART], first of all I started losing appetite then I came to a point where I would eat food and vomit immediately, then there is dizziness, I can’t concentrate on what I am doing, so it gave me a lot of problems.’* P2 facility L, male, age 37, on ART

*'The medicines I am getting, they have not caused me any problems… Most people complain a lot that the medicines sometimes treat them bad but for my case ever since I started this drug [ART] I have not been getting any problems related to my health.’* P5 facility G, female, age 26, on ART

Caregivers reiterated that patients experienced debilitating physical symptoms associated with HIV and its treatment:

*'She has been falling sick often, time and again she is down with malaria, fever, diarrhoea and general body pain and these days she gets severe pain in the bones and this pain has limited her from doing any other work.’* C4 facility G, female, age 40, patient’s friend

Symptoms were reported to interfere with patients’ physical function, sleep and ability to work.

b. Pain and symptom management

The benefit of receiving ART and pain and symptom control was a dominant theme across the facilities:

*'This service is prolonging the patient’s life. This is because that drug is now giving him more hope to live and as I said before, previously he was falling sick time and again. Now that he is taking the drugs the opportunistic infections are now few and because of this, he is doing other things even better than some normal people without the virus.’* C3 facility G, male, age 25, patient’s brother

However, problems were identified in relation to patients’ ability to access drugs, availability of drugs at the services, and staff-patient communication around pain. Logistical problems related to the high volume of patients seen at services were reported by patients and caregivers:

*'We queue for long when getting medicines, the people who are supposed to be serving us are just seated there and they are not attending to us. It takes such a long time that some people leave without their medicines.’* P3 facility C, male, age 37, on ART

Staff gave mixed reports about the availability of pain relieving drugs and other medication, reflecting the variability between the sites (see Table 1). Staff in Kenya discussed the unavailability of morphine and restricted access caused by regulations regarding prescription in outpatient settings:

'*At least we have the drugs that can control pain up to pethidine. The oral ones, maybe like morphine, are available but under prescription strictly. Those can be available but only in the inpatient [unit].’* S5 facility C, Doctor, 3 years’ experience

In Uganda, strong opioids were not available at three of the six sites:

*'What we don’t have is pain relief. We do not have strong opioids like morphine but [we] have Ephedrine [and] use weak opioids like ibuprofen, diclofenac, both [in] injection [form]–we have them but some strong opioids like morphine syrup we don’t have, but we have pethedine injection.’* S1 facility G, Nurse counsellor, 24 years’ experience

Even where services said they did have access, this could be variable:

*'I think it would be good to get oral morphine for pain management because we get certain patients in severe pain and all we have is codeine phosphate.’* S1 facility M, Clinical officer, 9 months’ experience

This quotation demonstrates that a lack of access to strong pain relieving drugs was usually recognised as a need by staff; however, this was not always the case, as demonstrated by a nurse counsellor in Kenya:

Interviewer: '*Is pain managed well?’*

Respondent: '*Yes… We have brufen.’*

Interviewer: '*What about cases of severe pain?’*

Respondent: '*We don’t have any other [medications] except brufen.’*

S6 facility A, 6 years’ experience

As well limitations in the availability of drugs and a need for staff training in pain management, barriers to communication of pain and other problems were also evident. Several patients and caregivers said that patients did not always report the pain they experienced to healthcare staff:

*'In fact, I don’t complain about these problems–take [as] an example this problem with my legs, I haven’t complained about it because I realised that they were not painful as a whole, but I mostly experience pains in the joint.’* P4 facility L, female, age 42, on ART

This lack of communication appeared to be related to patients’ perceptions of what staff were interested in and could help with.

Pain control was reported as more challenging in patients with advanced disease, in part due to lack of appropriate drugs (S4 facility J, Counsellor, 2 years’ experience). In Uganda, staff training on pain management and collaboration with local hospices was described:

*“First of all what we did was have a training for some of our staff on management of pain. This was conducted by [the local hospice] and we had clinical officer, nurses etcetera [who] we tried to follow and monitor on this treatment of pain in a larger manner.”* S5 facility G, Medical Superintendent, 5 years’ experience

Collaboration with and referral to the same hospice which conducted the training was reported to be useful by a nurse at a different service:

*“[For] severe pain, as I told you we work with [the local hospice. Sometimes they pay visits to us here when there is a pain [facility staff] can’t manage. We contact them and write referral letters and they come and visit our patients here.”* S2 facility L, Nurse, 8 years’ experience

Use of the World Health Organization’s (WHO) pain ladder [33] was described at one site where palliative care training had been provided:

*'We have a palliative care nurse and I think when someone is in severe pain the way you have called it then we consider morphine. She was trained and she usually gets the morphine and takes it to the clients. But usually [when] there is that severe pain they start with the usual pain killers like panadol, indocid–if the pain refuses to go then she results to morphine.’* S5 facility H, Nurse, 4 years’ experience

2. Psychosocial distress

a. Description of psychosocial distress

The psychosocial distress experienced by patients was a further dominant theme, with stigma described as a primary contributor:

*'If people out there get to know that one is HIV positive, they treat you badly. At times if you are employed you may lose your job if you disclose your status. Even in the family, if you disclose that you are HIV positive, your people may stop eating with you, they just ignore you saying that you are useless since you can die any time. You can even be denied a scheme loan because of your sero status–they claim that you can die any time and therefore default.’* P2 facility M, male, age 41, on ART

Caregivers also suffered from the effects of stigma:

*'What hurts me is the way other people look at us–they say that my husband was a proud man so now it is his time to face the problems, so when I hear those words I feel so hurt.’* [Caregiver breaks down in tears] C1 facility H, age 35, patient’s wife

Patients and caregivers described how stigma exacerbated isolation and loneliness, which could have a negative impact on adherence:

*'Usually you find that these patients have withdrawn away from the community and their close people. They just stay alone and give up and lose hope in life and also feel that they have already gone to the end of life… because of this negative thinking towards life usually they don’t come for medication’* C2 facility G, age 24, patient’s brother

The suffering and 'psychological torture’ (P6 facility L, female, age 40, on ART) caused by poverty was also highly evident in patients’ and caregivers’ accounts, manifested in worries regarding having enough food to eat and money to pay for transport to collect treatment or children’s education:

*'The [biggest] problem that I have had is that of food. I used to live with my sister and I don’t get along with her. When she heard that I have the HIV virus, she chased me away. I now live with other ladies and I don’t work.’* [Starts to cry] P3 facility D, female, age 24, on ART

Poverty had a detrimental impact on patients’ adherence to ART:

*'Sometimes you cannot afford to buy food to eat; at times you cannot sustain yourself completely. At times you cannot afford transport costs to come for treatment. And again with these drugs we take, one must feed well and because you cannot really work hard enough because of compromised health at times even getting this food is a big problem. So you may have to stop the drugs because they really raise your appetite.’* P1 facility L, male, age 37, on ART

Staff highlighted patients who were newly diagnosed or had acquired HIV at a young age as particularly susceptible to psychological distress, and this was supported by the patient data:

*'One thing that has really brought grief and pain to me is that I acquired HIV at an early age before even having a child. That is what hurts me.’* P3 facility K, female, age 26, on ART

b. Management of psychosocial distress

Staff discussed the psychological difficulties facing patients and the counselling and peer support offered to alleviate these problems:

*'[We encourage patients] not to lose hope and know that life still goes on, know that this isn’t the end, and you can even draw examples from people who have lived with this infection for a long time, so that eventually she sees herself not alone in the ocean… Usually we use the PWAs [people living with AIDS] because they’ve gone through it.’* S1 facility J, Counsellor, 10 years’ experience

The benefit of counselling, particularly during home visits, was reiterated by patients and caregivers:

*'The good thing is that for any infection or illness he is treated, he is also given counselling so that he loves himself despite having HIV.’* C1, facility H, age 35, patient’s wife

*'Some counsellors come and visit us from home, but not all the counsellors are doing that, but some of them do take time and visit their clients or at the place he knows where you are staying, asking you how are doing, your children, what’s your problem, what are you doing, such things.’* P2 facility H, male, age 45, not on ART

However, as with physical pain, patients’ social and psychological needs were not necessarily shared with healthcare staff:

Interviewer*: 'Are there any other problems that you have [which you] have not been able to talk about with the healthcare workers?’*

Respondent: '*Other problems include where we live; both the roof and walls of those houses are made of corrugated iron sheets and the floor is made of earth. With the cold Kericho weather, the problem of beddings is quite real to me… because I do not have a lot of money; I can only buy the blanket that costs 200 [Kenyan] shillings and this is very light.’* P1 facility E, male, age 40, on ART

Compounding the problem, where social needs were raised with staff, not all patients reported positive experiences:

*'They tell us that they can’t discuss with us social issues, and that their work is to give us medicines They ask us whether we want to get well or to talk about social problems… Once in a while, even when you try to tell them something they tell you that it is not their problem and they go away.’* P7 facility A, female, age 45, on ART

Some services, particularly in Uganda, offered food and transport costs to patients and families, but this was not always sustainable, and a strategy of phasing out support for patients was described:

*'Most patients have a cry that they have been phased out as beneficiaries of food support… According to the hospital management they do it because it is supposed to be an emergency support to the most vulnerable patients. They do it in order to support other new patients… I would have wished this support to continue, because most of the patients are too weak and it is going to be difficult for them to survive without this service.’* C2 facility G, age 24, patient’s brother

Other ways of providing support were also reported, including kitchen gardening initiatives and cooperative income generation. One man, who was running a small shop selling vegetables he had grown, explained how the service had helped him:

*'[Service staff] advise[d] us to make a group of around 10 people and contribute some little money as members concerned, so that when you are in need you go there then get something, or else [they] just teach us some skills–farming, bead making, hand crafts–so that when you are in need of money you either sell cabbages, tomatoes or you just sell your hand crafts as they taught you.’* P5 facility H, age 38, not on ART

3. Spiritual distress

a. Description of spiritual distress

Illness was often experienced by patients through the lens of their spiritual or religious beliefs:

*'After some time I started getting serious drug side effects* [interviewee breaks down in tears]*. I could not overcome the drug side effects and I was tempted to stop, probably by an evil spirit. But with prayers I continued taking the drugs.’* P7 facility G, female, age 56, on ART

Although the supportive role of spiritual practices and beliefs was described, patients experienced existential despair and hopelessness and questioned God:

*'My biggest worry is about my future. I have no child and my dreams are shuttered down and it hurts lots. It is an innermost pain which I can’t explain to anybody. When you see people who have died because of this disease and yet they have been on ARVs and yet they die mysteriously... I feel weighed down and lose hope most often*.’ P3 facility L, male, age 36, on ART

This was reiterated by staff:

*'At times when people get sick you realise that there is that tension or tendency of saying that God is away from me because if it were true that God is alive then he would not have let me fall sick in the way that I am. So you will find that there is that kind of antagonism between him and his God.’* S2 facility H, Counsellor, 2 years’ experience

Patients and caregivers also described the impact of illness on patients’ spiritual lives:

*'He may be having a skin rash so he cannot go to church for people to look at him and stigmatise him… there is that problem of stigma and stress… This is a big challenge for men: once he sees his skin has a rash, he has lost his hair, they tend not to want to join other groups or even go to church for prayers.’* C5 facility H, age 26, patient’s brother

b. Management of spiritual distress

Some patients and caregivers described a spiritual element to the counselling received from the facility’s healthcare workers:

*'[The healthcare workers] encourage us to keep on going to church and they tell us to have hope. They tell us that an HIV diagnosis does not mean this is the end of the world… they tell us to keep with our religious leaders. Moslems keep in touch with mosques, Christians keep in touch with the church.’* P1 facility A, male, age 49, not on ART

The perceived benefits of spiritual support were described by a caregiver at facility G, a missionary hospital:

*'Spiritual counselling helps us because it brings everyone (healthcare worker, patient and caregiver) closer to God. For example, when you know God it harmonizes the relationship among all these parties involved and humbles the patient. When a patient is humbled he is loved more and can easily be helped by people around him. And if you don’t have that touch with God you might think that people don’t know what you are going through.’* C3 facility G, age 25, patient’s brother

Some nurses, social workers and counsellors described spiritual care as part of their role, but at several facilities, particularly in Kenya, spiritual care seemed to be rarely offered:

Interviewer: *'Do they ever talk to you about religion, spiritual matters?’*

Respondent: *'No [laughs], we only talk about clinical matters. I haven’t heard anything to do with spirituality.’* P1 facility D, female, age 41, on ART

Staff suggested this could be due to limited space and time, patients’ own preferences or staff fears of tackling this sensitive area:

*'It should be the healthcare workers job, but we lack the skills and time. Sometimes people consider it to be very sensitive and they just leave it out. People do not want to disclose private things and you must watch your move if you want to talk about spirituality and beliefs.’* S4 facility M, nurse, 1 year’s experience

The challenges of caring for a patient with potentially harmful spiritual beliefs were described by several staff members, and this was also offered as justification for avoiding discussion of spiritual matters:

*“I don’t touch much about spiritual care because there is a controversy between HIV and spiritual care. Many clients we have come across, they will tell you, 'I went to be prayed for, I went to pastor so and so, I paid some 3000 or 4000 [Kenyan] shillings or I didn’t pay anything; I stayed with him for 24 hours, they prayed for me and am now healed.’… They can even quote that, 'There’s a friend of mine, there’s a neighbour who came and attended pastor so-and-so’s church and when they came back…’ So I tell them go to whatever church they want to go to, but remember, your status is the same.”* S4 facility D, Nurse, 4 years’ experience

## Discussion

This is one of the first studies to explore the multidimensional problems experienced by HIV outpatients in sub-Saharan Africa and how these are managed by outpatient facilities. The study has advanced knowledge of this population by taking a person-centred, multi-perspective approach to explore the domains of wellbeing stipulated in policy guidance, while going beyond prior single-domain studies to describe the interrelatedness of these domains.

Findings highlight the stark reality of living with HIV, confirming that patients experience psychosocial and spiritual suffering, as well as physical pain and other symptoms. The use of multiple perspectives enabled triangulation of findings, contributing to validity. Patients’ everyday lives were characterised by poverty and stigma: they were preoccupied with worries about basic needs such as food, employment and transport to collect medication, and described feelings of isolation and experiences of discrimination which added to the burden of living with HIV. The existential impact of HIV, including hopelessness, fears of the future and feelings of despair and doubt, was intimately related to psychosocial and physical suffering.

The findings elucidate the detrimental effect of stigma on patients and their families. Stigma against those with HIV is highly prevalent in sub-Saharan Africa [34,35]. In collectivist societies in which social relationships are highly important [36], the experience of stigma and social isolation may be particularly detrimental. Stigma contributes to non-adherence to ART [37,38] and is associated with rejection [39], breakdown of social support [39,40], difficulty finding work [41] and poor mental health [19].

Pain management at the facilities was limited by problems with opioid availability. As in Dekker et al’s study of a public hospital and its clinics in the Eastern Cape, South Africa, strong pain medicines were often in short supply or unavailable [42]. Dekker et al. found that health care providers’ misperception of palliative care as end of life care (and hence inappropriate to patients with HIV) constituted a barrier to adequate pain control in HIV patients. Findings from our study provide further support for the need for staff training in the palliative care approach, including psychosocial and spiritual support as well as improved pain control. Counselling and limited spiritual support were described, but unmet psychosocial and spiritual needs appeared to contribute to and exacerbate patients' experience of pain. The findings from this study thus support the notion that pain is a complex phenomenon which can have nonphysical as well as physiological dimensions and causes, as captured in the concept of 'total pain’ [42]. The total pain model recognises that pain is multifaceted and that psychological, social and spiritual problems can contribute to the overall phenomenology of pain [42]. While previous research into the experience of total pain has focussed on developed country settings [43-45], this study demonstrates the total pain experienced by HIV patients in sub-Saharan Africa.

**Figure 1:**
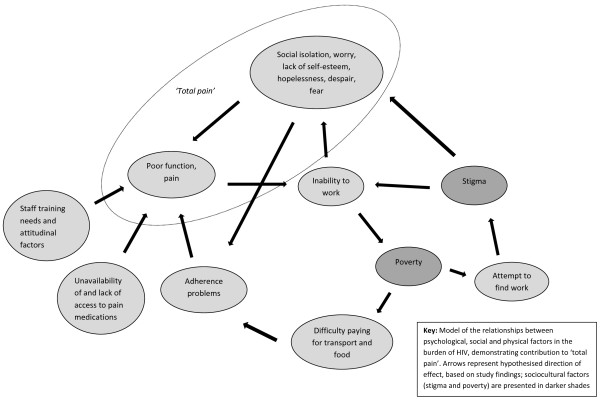
The HIV burden cycle.

Relationships between physical, psychosocial and spiritual dimensions of burden were evident; for example, difficulty paying for food and transport and the consequences of stigma (isolation, a lack of social support, low self-esteem and loneliness) could all contribute to poor adherence to treatment regimens. Findings regarding the interconnections between psychosocial and physical factors in the experience of HIV are modelled in Figure 1.

This model demonstrates the imperative to tackle poverty and stigma to alleviate psychosocial distress and support adherence, and the need to consider the wider sociopolitical context in which HIV care is provided. Patients in this study required multidimensional care rather than care focusing solely on the management of physical pain and symptoms, supporting the mandate to provide palliative care alongside active treatment of HIV. Patients in this study did not always report their pain and suffering to facility staff, demonstrating the importance of regular assessment of multidimensional problems [46].

The study findings also support evidence from epidemiological studies regarding the negative impact of poverty on adherence to ART. The costs of drugs, transport, opportunity costs such as having to forgo a day’s pay [24,37,47] and lack of adequate food security (as some medications can only be taken on a full stomach) [48] contribute to non-adherence to ART. Hunger and malnourishment further compromise the immune systems of people with HIV, diminishing the body’s ability to fight infection [49], and making adherence to complex ART regimes difficult or impossible [50].

There are a number of limitations to this study which should be born in mind when considering the findings. Translating the transcripts into English rather than conducting the analysis in local languages means that nuances in meaning may have been lost. However, collecting data in local languages and the robust translation procedure ensured high conceptual accuracy. The sampling used in this study was not purposive but rather based on selecting the largest organisations from a random sample of PEPFAR services. This may have resulted in a sampling bias, and the individual characteristics of services should therefore be considered in determining the transferability of findings.

### Clinical and policy recommendations

The findings have four main implications for the provision of HIV care in sub-Saharan Africa and in other developing country settings with high rates of HIV infection. First, HIV outpatients require holistic care that responds to physical, psychological, social and spiritual care needs in line with palliative care philosophy, whether or not they are on ART [51,52]. Collaboration between hospitals, clinics and hospices and increased access to palliative care training for staff is likely to be effective in this regard [42]. The burden cycle (Figure 1) provides a model to guide such care, subject to further testing. There is a need for training and support for informal caregivers and for interventions to enhance economic and employment opportunities of patients with HIV, in order to improve quality of life and reduce caregiver burden [53]. Uganda was the first country in Africa to have made palliative care for people with HIV and cancer a priority in its National Health Plan (2000-2005) [54] and one of the 49 medical services designated as 'essential clinical care’ [55], and serves as an example to other countries in this regard. In Kenya, although there has been some progress in palliative care provision, more remains to be done, particularly towards improving access to medication for moderate to severe pain and developing a plan of action for palliative care integrated with HIV care [56].

Second, continued advocacy to ensure the availability of pain-relieving drugs, including opioids, is essential [23]. Morphine and codeine should 'be available within the context of functioning health systems at all times in adequate amounts, in the appropriate dosage forms, with assured quality, and at a price the individual and the community can afford’ [57].

Third, the fact that pain, whether physical or psychosocial in nature, was not always reported to healthcare staff, means routine assessment embedded in clinical practice is required as standard. Proactive questioning to ascertain patient needs may be facilitated by communication skills training for staff as well as use of the APCA African Palliative Outcome Scale in clinical practice [58].

Fourth, community initiatives to continue to reduce stigma and discrimination against those with HIV infection and their family members are required. There is evidence that such initiatives should involve debate and dialogue to challenge obstacles to changing health-damaging attitudes and behaviours [34,59].

### Research recommendations

The model presented in Figure 1 should be subjected to further testing in other African HIV populations and using quantitative methods.

The effectiveness of palliative care interventions for HIV patients in sub-Saharan Africa should be determined. A systematic review of the effect of palliative care on HIV patient outcomes found that home palliative care and inpatient hospice care significantly improved outcomes in the domains of pain and symptom control, anxiety, insight and spiritual wellbeing [50]. However, only five papers from Africa were identified, and none of these reported a quantitative evaluation of the outcomes of palliative care. Evaluation and outcome data are essential in developing country settings where best use must be made of available resources [24,60].

Lastly, there is some evidence that psychological support in the form of peer support groups may be effective in reducing mental disorder in African HIV populations [19], but further research is required to establish good practice in the provision of psychosocial and spiritual support to patients with HIV in sub-Saharan Africa.

## Conclusions

This study highlights the multifaceted nature of the burden experienced by HIV outpatients and the ways in which HIV outpatient services respond to this burden. The findings suggest that HIV healthcare providers need to identify and respond to psychosocial and spiritual dimensions of distress in conjunction with ensuring the excellent management of pain and other symptoms. Patient problems are interrelated, therefore assessment and treatment should be in line with a person-centred, holistic paliative care approach that reflects patients’ self-reported needs. This kind of approach to care is likely to result in better health outcomes in this population.

## Competing interests

The authors declare that they have no competing interests.

## Authors’ contributions

LS conceived of and conducted data analysis and wrote the paper. VS and PS helped design the study, managed data collection, contributed to data analysis and commented on the paper. RP, FMP and JD helped design the study, oversaw data collection, contributed to data analysis and commented on the paper. GM and NG collected the data, contributed to analysis and commented on the paper. RH and IJH conceived of the study, obtained funding, managed the study and contributed to the paper. All authors read and approved the final manuscript.

## Pre-publication history

The pre-publication history for this paper can be accessed here:

http://www.biomedcentral.com/1472-684X/12/35/prepub
